# Nanosized CoO Loaded on Copper Foam for High-Performance, Binder-Free Lithium-Ion Batteries

**DOI:** 10.3390/nano8040183

**Published:** 2018-03-22

**Authors:** Mingna Liao, Qilun Zhang, Fengling Tang, Zhiwei Xu, Xin Zhou, Youpeng Li, Yali Zhang, Chenghao Yang, Qiang Ru, Lingzhi Zhao

**Affiliations:** 1 Institute of Opto-Electronic Materials and Technology, South China Normal University, Guangzhou 510631, China; lmn0769@163.com(M.L); aaron2222222@outlook.com (Q.Z.); zxallin@163.com (X.Z.); youpli@163.com (Y.L.); zhangyalicherish@163.com (Y.Z.); 2 Guangdong Provincial Engineering Technology Research Center for Low Carbon and Advanced Energy Materials, Guangzhou 510631, China; 3 Department of Medical Engineering, Guangdong Food and Drug Vocational-Technical School, Guangzhou 510663, China; tang15626408613@163.com; 4 Guangzhou Zhixin Middle School, No.152, Zhixin South Road, Guangzhou 510080, China; lmn0753@163.com; 5 New Energy Research Institute, School of Environment and Energy, South China University of Technology, Guangzhou 510006, China; esyangc@scut.edu.cn

**Keywords:** CoO nanoflakes, CoO nanoflowers, anodes, binder free, lithium ion batteries

## Abstract

The synthesis of nanosized CoO anodes with unique morphologies via a hydrothermal method is investigated. By adjusting the pH values of reaction solutions, nanoflakes (CoO-NFs) and nanoflowers (CoO-FLs) are successfully located on copper foam. Compared with CoO-FLs, CoO-NFs as anodes for lithium ion batteries present ameliorated lithium storage properties, such as good rate capability, excellent cycling stability, and large reversible capacity. The initial discharge capacity is 1470 mA h g^−1^, while the reversible capacity is maintained at 1776 m Ah g^−1^ after 80 cycles at a current density of 100 mA h g^−1^. The excellent electrochemical performance is ascribed to enough free space and enhanced conductivity, which play crucial roles in facilitating electron transport during repetitive Li^+^ intercalation and extraction reaction as well as buffering the volume expansion.

## 1. Introduction

Over the past few decades, lithium-ion batteries (LIBs) have been widely used as a predominant energy transformation and storage system for laptops, electric vehicles, and portable electronic products [1,2,3]. Nevertheless, commercial carbonaceous anodes are limited by their low capacity (~372 mA h g^−1^), which means they can barely meet the growing requirements for next-generation LIBs [4,5]. To solve this problem, a worldwide effort has been made to explore alternative anode materials with higher power density and energy density [6,7,8,9,10]. The transition metal oxides (TMOs) such as tin, nickel, and iron oxides have shown almost three times the capacity of commercial graphite [11,12,13,14]. Therefore, CoO has been extensively researched owing to its high theoretical capacities (716 mA h g^−1^) based on the conversion mechanism and its completely reversible electrochemical reaction as follows: $\mathrm{CoO}+2{\mathrm{Li}}^{+}+2e^{-}↔{\mathrm{Li}}_{2}O+\mathrm{Co}$, which is different from the reaction of lithium intercalation and de-intercalation in a carbon anode: $6C+x{\mathrm{Li}}^{+}+xe^{-}↔{\mathrm{Li}}_{x}C_{6}$ [15,16,17]. Nevertheless, the drastic volume expansion effect and poor electrical conductivity restrict the improvement of CoO’s electrochemical performance [18,19,20,21,22]. Up to now, a great deal of work has been done to overcome the above issues by designing various specific nanostructures such as nanospheres [23], nanocubes [24], nanowires [25], nanosheets [26], etc. Although these efforts magnify the interface area of electron/electrolyte and increase reactive sites, they barely promote the electrical conductivity of the electrodes owing to the poor ion/electrical transport kinetics shown by metal oxides [27].

Recently, several research groups have synthesized binder-free anodes by growing various CoO nanostructures directly on the current collector substrate. This is a way to avoid introducing PVDF (Polyvinylidene Fluoride) and carbon black into the anodes. Through this method, no extra weight will be added to the electrodes. What is more, the removal of PVDF can improve the overall electrical conductivity and specific capacity. It has been reported that CoO nanostructures grown on various conductive substrates such as copper foil [28], nickel foam [29], Ti foil [30], and carbon cloth [31]. Huang et al. synthesized the CoO/graphene hybrid on the copper foil by electrostatic interaction and the composite delivered 947 mA h g^−1^ capacities in the first cycle at 100 mA g^−1^ [32]. By preparing CoO nanowires on Cu foil through hydrothermal synthesis, Cao et al. fabricated an electrode whose reversible specific capacity could be sustained at 1248.8 mA h g^−1^ [33]. Zhao et al. used a hydrothermal method to grow hierarchical CoO nanowires on carbon cloth that delivered a reversible capacity of 1300 mA h g^−1^ at 100 mA g^−1^ after 90 cycles. However, the capacity of the electrode dropped to 600 mA h g^−1^ after the current density increased from 100 to 2000 mA g^−1^ [31]. The above studies made improvements to the electrochemical performance of CoO to some degree; however, there is still room for improvement of the reversible capacity and cycling performance.

**Scheme 1:**
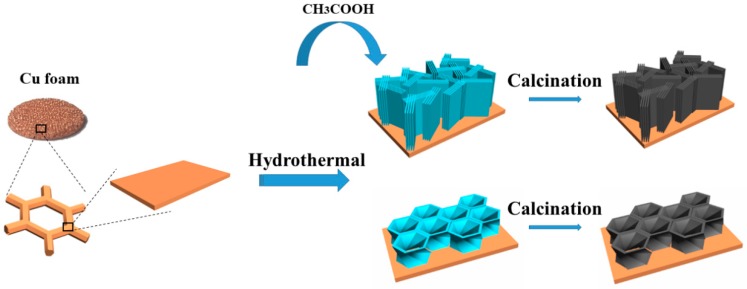
Schematic illustration of the synthesis process of CoO with two different morphologies on copper foam substrates.

In this paper, we fabricated different nanosized CoO by a simple hydrothermal method, including nanoflakes and nanoflowers on copper foam, and directly applied them as anodes for LIBs (shown in Scheme 1). As the temperature rises in the oven, the urea hydrolyzed and released CO_3_^2−^ and OH^−^ anions. The formation of a cobalt–hydroxide–carbonate nucleus started, along with the increase in the concentration of anions in the reaction solution. After the annealing process, the precursor was decomposed completely and CoO electrodes were obtained. The overall chemical reactions are expressed as follows: ${\mathrm{Co}}^{2+}+0.5\left(2-x\right){\mathrm{CO}}_{3}^{2-}+x{\mathrm{OH}}^{-}+nH_{2}O\to \mathrm{Co}{\left(\mathrm{OH}\right)}_{x}{\left({\mathrm{CO}}_{3}\right)}_{0.5\left(2-x\right)}\cdot nH_{2}O$; $\mathrm{Co}{\left(\mathrm{OH}\right)}_{x}{\left({\mathrm{CO}}_{3}\right)}_{0.5\left(2-x\right)}\cdot nH_{2}O\to \mathrm{CoO}+0.5\left(2-x\right){\mathrm{CO}}_{2}+\left(n+x/2\right)H_{2}O$. As expected, both CoO-NFs and CoO-FLs displayed a superior rate capability and high reversibility capacity. A comparison of the performance of CoO-NFs in this work and the reported CoO anodes with different morphologies is shown in Table 1.

The considerable improvement in performance is mainly owing to the following factors. Firstly, the unique morphologies provide good strain accommodation for the significant volume change of CoO within the Li^+^ extraction and insertion process. Secondly, the uniform and thin CoO growing in situ on copper foam can shorten the charge transfer pathways, causing a faster electronic diffusion. Thirdly, copper foam as a current collector can avoid the adverse influence of PVDF binder with poor electrical conductivity. Moreover, there are rare reports on synthesizing nanosized CoO directly on copper foam through a facile hydrothermal route.

## 2. Experimental

### 2.1. Material Preparations

In this work, all the analytical purity reagents were used without further purification. The copper foam, cut into a circular shape 14 mm in diameter, was washed by sonication sequentially in diluted acid solution, acetone, and ethanol separately for 10 min. Typically, 30 mL deionized water were used to dissolve 0.2343 g CH_3_COOCo·4H_2_O and 0.5727 g urea at room temperature under magnetic stirring. The pH value of the mixture was adjusted to 4 by adding ~0.2 mL 3 mol/L CH_3_COOH solution with 30 min stirring to produce a uniform solution. Then the prepared copper foam was immersed into the mixed solution and transferred into a 50-mL Teflon-lined stainless steel autoclave. After being heated for 16 h at 120 °C, the sealed autoclave naturally cooled down. The substrates were washed with ethanol and deionized water to remove the residual reactants. After being dried for 12 h at 60 °C in a vacuum oven, the obtained precursors were annealed at a slow heating rate of 2 °C/min at 450 °C for 4 h in a nitrogen atmosphere. The neutral sample was synthesized by following the process given above without adding COOH. The corresponding samples were denoted as CoO-NFs (acidity) and CoO-FLs (neutral), respectively.

### 2.2. Material Characterizations

The chemical constitutents of the samples were evaluated by X-ray photoelectron spectroscopy (XPS, Axis Ultra DLD, Kratos, Shimadzu, Japan) and an X-ray diffractometer (XRD, BRUKER D8 ADVANCE, Cu Kα1: 1.5406, Billerica, MA, USA). The morphologies and structures of the synthesized products were determined by scanning electron microscopy (FESEM, 5 kV, ZEISS Ultra 55, Pt-spraying treatment, Oberkochen, Germany) and transmission electron microscopy (TEM, 200 kV, JEM-2100HR, JEOL Ltd., Beijing, China). ICP-OES analysis was evaluated by an inductively coupled plasma-optical emission spectrometer (SPECTRO ARCOS ICP-OES analyzer, SPECTRO, Kleve, Germany). The pore distribution and the specific surface area of the products were characterized by the Brunauer–Emmett–Teller area measurement (BET, Micromeritics ASAP 2020, Micromeritics, Shanghai, China).

### 2.3. Electrochemical Measurements

Galvanostatic charge/discharge (GC) measurements were performed on the LAND cell test system (Land CT 2001A, Sanland, Wuhan, China) using CR2032-type coin cells. Cyclic voltammetry (CV) measurements and impedance spectra were carried out with an electrochemical workstation (CHI 660E, Chenhua, Shanghai, China) in the frequency range from 0.01 Hz to 10 kHz. For comparison, coin cells were assembled to investigate the electrochemical properties of CoO-NFs and CoO-FLs. The synthesized CoO-NFs and CoO-FLs, as binder-free anodes, were used directly for electrochemical tests. The counter and reference electrodes were lithium foil, while the separator was polypropylene membrane (Celgard 2400). 1 M LiPF6 dissolved in a 1:1 (vol.) mixture of diethyl carbonate (DEC) and ethylene carbonate (EC) was used as the electrolyte. An argon-filled glove box (LG1200/750TS, Vigor, Suzhou, China) was used to assemble the CR2032-type coin cells.

## 3. Results and Discussion

### 3.1. Structural and Morphological Characterization

To estimate the loading mass of CoO on copper foam, the result of the ICP-OES test is displayed in Table 2. The samples of CoO-NFs and CoO-FLs were prepared using the same process. Therefore, the mass loading of CoO is estimated according to Equation (1) and the average of the results is used for the electrochemical measurements.

(1) $$m_{CoO}=\frac{m_{Co}\times M_{CoO}}{M_{Co}}$$

**Figure 1:**
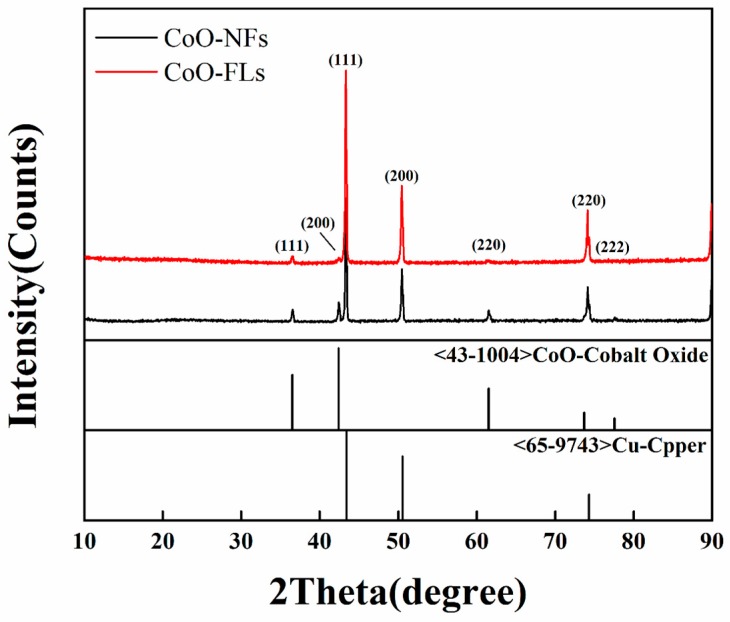
XRD patterns of CoO-NFs and CoO-FLs.

The crystalline phases of CoO-NFs and CoO-FLs are confirmed with the XRD patterns (Figure 1). The peaks of CoO-NFs are similar to CoO-FLs. Diffraction peaks located at 36.50°, 42.40°, 61.52°, 73.70°, and 77.56° match very well with the (111), (200), (220), (311), and (222) planes of the standard CoO phase (JCPDS No.43-1004). Moreover, additional diffraction peaks at 43.4°, 50.5°, and 74.3° correspond to the (111), (200), and (220) planes of copper foam (JCPDS No. 65-9743). The characteristic peaks of CoO-NFs are stronger and sharper than CoO-FLs, which manifest better crystallinity and higher crystalline purity than CoO-NFs.

**Figure 2:**
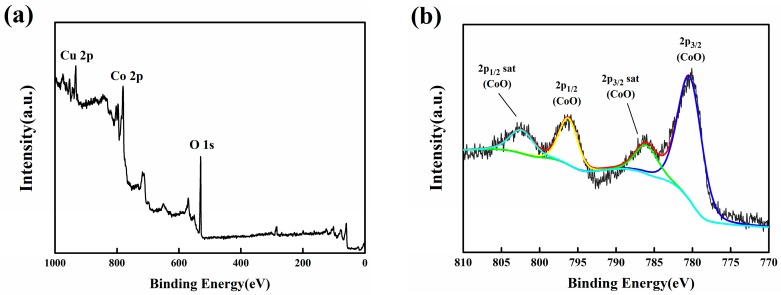
XPS survey spectrum (**a**) and spectra of Co 2p (**b**) performed on CoO-NFs.

To further confirm the chemical compositions, Figure 2a shows the CoO-NFs detected by X-ray photoelectron measurement. The XPS wide survey spectrum verifies the presence of elemental Cu, Co, and O in CoO-NFs. The Co 2p spectrum is shown in Figure 2b. The peaks at 780.1 eV and 796.4 eV are assigned to Co 2p3/2 and Co 2p1/2, respectively. Furthermore, the peaks at 786.1 eV and 802.8 eV are associated with Co 2p3/2 and Co 2p1/2 shakeup satellite, respectively [36,37,38]. The Co 2p pattern suggests a divalent cobalt oxidation state, displaying the formation of a Co–O bond in CoO-NFs.

**Figure 3:**
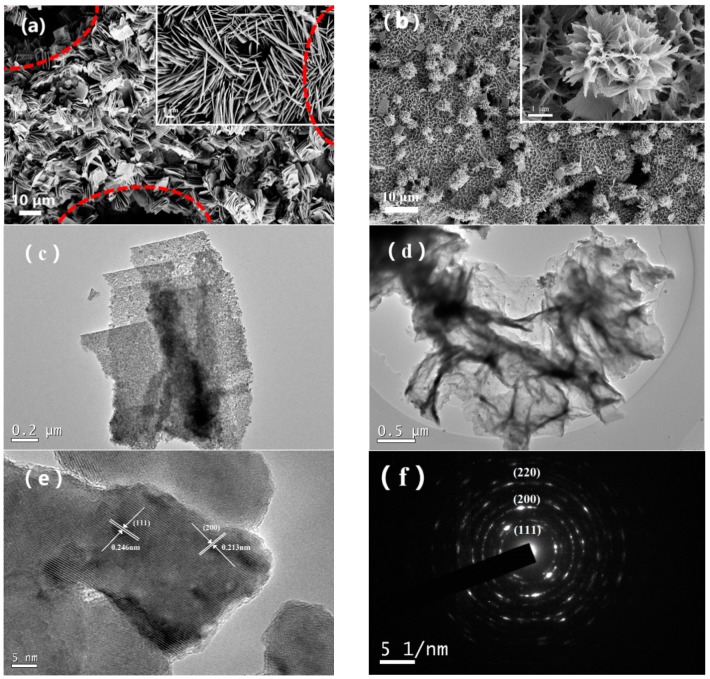
SEM images of CoO-NFs (**a**) and CoO-FLs (**b**); TEM images of CoO-NFs (**c**) and CoO-FLs (**d**); HRTEM image (**e**); and the selected area electron diffraction (SAED) pattern (**f**) of CoO-NFs.

Next we investigated the morphologies of as-prepared nanosized CoO electrodes. In the low-magnification pattern, as shown in Figure 3a, reticular copper foam covered with many flakes can be observed. The magnified pattern (inserts in Figure 3a) exhibits dozens of nanoflakes uniformly distributed on the substrate; the thickness of the flakes is just a few tens of nanometers. These interlaced nanoflakes have a regular vertical arrangement and offer enough free space and a large surface area, which is beneficial to ion/electron transport. Figure 3b and the inset show the low and high magnification SEM patterns of CoO-FLs. In the low magnification pattern, CoO clusters with a flower-like structure can be observed. The CoO flakes grew not only on the surface, but also suspended over the cavities of copper foam. Without the support of copper foam, the CoO-FLs anode was vulnerable to structural damage during the lithium ion transport process. The morphology and structure of CoO-NFs and CoO-FLs were investigated by transmission electron microscopy in Figure 3c–f. As observed, the morphologies of CoO-NFs and CoO-FLs are in coordination with the SEM images exhibited above. Figure 3c shows that CoO-NFs are composed of numerous interconnected nanoparticles, manifesting porous architecture. This unique configuration can not only conveniently facilitate the infiltration of electrolyte into the electrode but also maintain structural stability. In contrast, CoO-FLs have the characteristic of a smooth and compact surface, shown in Figure 3d, which would impede the immersing of the electrolyte and the diffusion of lithium ions. The high-resolution TEM image of CoO-NFs in Figure 3e shows two kinds of clear and continuous fringes in the same orientation, with lattice spacing of 0.213 and 0.246 nm, which correspond to the (200) and (111) planes of CoO-NFs, respectively. The structure of CoO-NFs was further confirmed by the electron diffraction pattern in the same area, as shown in Figure 3f. The three diffraction rings are ascribed to the (220), (200), and (111) planes [39], which have a mutually verifying relationship with the analysis of XRD pattern (Figure 1).

### 3.2. Electrochemical Performances

**Figure 4:**
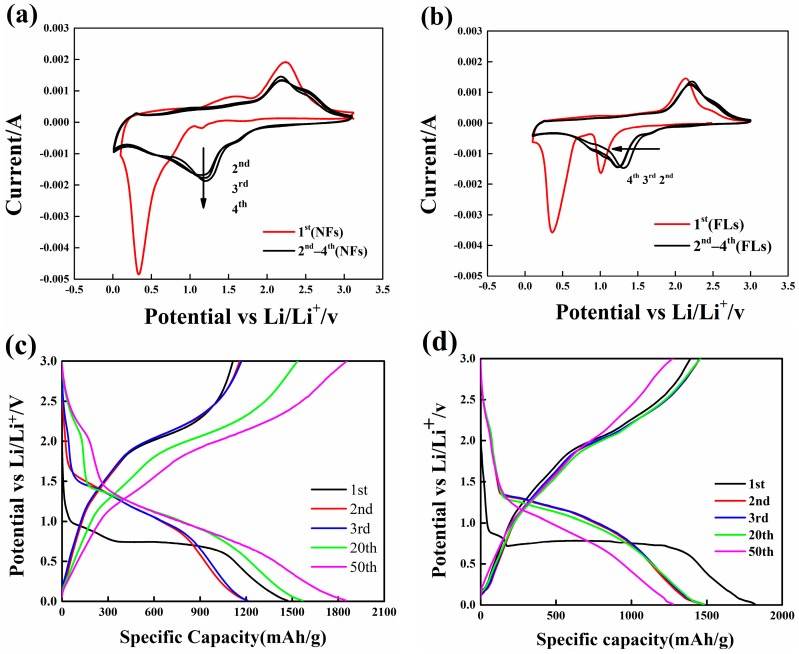
CV curves for the first four cycles of CoO-NFs (**a**) and CoO-FLs (**b**) at a scan rate of 0.2 mV s^−1^; Discharge and charge voltage profiles of the CoO-NFs (**c**) and CoO-FLs (**d**) at a current density of 100 mA g^−1^.

To demonstrate the electrochemical performances of CoO-NFs and CoO-FLs, the cyclic voltammetry was carried out in a voltage range of 0.01–3.0 V at a slow scan rate of 0.2 mV s^−1^ (Figure 4a). Cathodic peaks in the first cycle at ~0.35 V are found in both CoO-NFs and CoO-FLs, corresponding to the formation of the solid electrolyte interface film, while cathodic peaks at 1.0–1.2 V are ascribed to the reduction of CoO into Co [40,41]. In the anodic process, therefore, the oxidation peaks of both CoO-NFs and CoO-FLs that appear at 2.25 V can be associated with the decomposition of Li_2_O. In the second cycle, the cathodic peaks shifted to 1.25 V because of the amorphization of CoO and the formation of ultrafine nanograins during the first cycle [42]. Figure 4b exhibits the discharge/charge curves of CoO-NFs at 100 mA g^−1^ current density. The first discharge profile has a long plateau at about 0.75 V, which is ascribed to the formation of the SEI films and the complex phase transformation of CoO to Co. This electrochemical reaction can be described as in Equation (2). The first charge profile displays a strong polarization at 1.4 V. There is a voltage plateau at 2.25 V, corresponding to Equation (3). Then the plateau shifts to higher voltage at ~1.5 V in the following discharge profiles.

(2) $$\mathrm{CoO}+2{\mathrm{Li}}^{+}+2e^{-}\to \mathrm{Co}+{\mathrm{Li}}_{2}O$$

(3) $$\mathrm{Co}+{\mathrm{Li}}_{2}O\to \mathrm{CoO}+2{\mathrm{Li}}^{+}+2e^{-}$$

The voltage hysteresis exhibited in the following cycles is a general phenomenon for the transition metal oxide Li-ion batteries’ anode materials and could be noted as the signature of the conversion mechanism [43]. The CoO-NFs deliver 1113 mA h g^−1^ and 1470 mA h g^−1^ for charge and discharge capacity, respectively. The high surface area of the nanostructure of the anode not only increases the reactive sites of the electrode/electrolyte, but also increases the Li^+^ consumption resulting from the SEI (solid electrolyte interface) formation. Moreover, a series of side reactions at initial cycles such as the reduction of the metal oxide, the decomposition of the electrolyte, and the reaction of the oxygen-containing functional groups on the metal oxide surface with Li^+^ is also responsible for the irreversible capacity [44]. Moreover, the sloped plateaus of discharge at ~0.8 V and ~1.25 V during the following cycles are in coordination with the cyclic voltammetry result.

**Figure 5:**
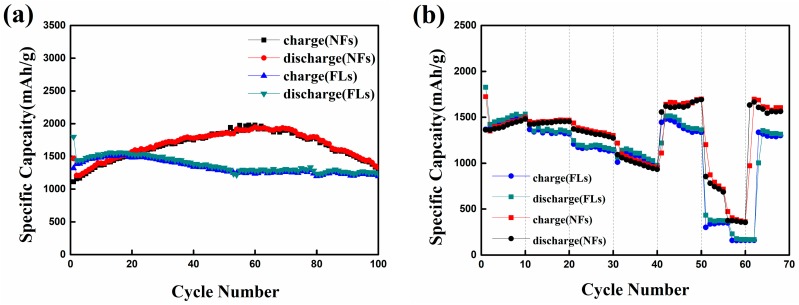
(**a**) Comparative cycling performance of CoO-NFs and CoO-FLs at 100 mA g^−1^; (**b**) the rate capability of CoO-NFs and CoO-FLs at different current densities.

Figure 5a shows the cycling performance of CoO-NFs and CoO-FLs at room temperature at 100 mA g^−1^ current density. As shown in the picture, CoO-NFs and CoO-FLs exhibit high initial and reversible capacity during 80 cycles. The copper foam, as a current collector, not only extended the electron/electrolyte interface but also promoted the conductivity of the full electrodes. The initial coulombic efficiencies of CoO-NFs and CoO-FLs are 75.7% and 73.4%, respectively. This may result from the large surface area of two samples so that more irreversible Li^+^ was consumed in the formation of SEI films. The coulombic efficiency increases to ~96% (in the second cycle) and the tendency is upward in the following cycles. Researchers have observed a similar capacity rise phenomenon during cycling in transition-metal oxide composite anode materials [45,46]. The dominant factor in the increase in capacity can be regarded as the reversible growth of polymer/gel-like films [46,47]. In the process of conversion, the polymeric films can be ascribed to kinetically active electrolyte degradation [48]. After 60 cycles, the cycling performance of the two samples gradually becomes stable. The reversible capacity is 1776 mA h g^−1^ after 80 cycles, which is significant improvement compared with the reported CoO-Ni foam electrode [34]. The rate capability of CoO-NFs and CoO-FLs is tested at a series of rates to estimate their electrochemical performance. As shown in Figure 5b, the average discharge capacities of CoO-NFs are 1459, 1457, 1353, 1038, 868, and 494 mA h g^−1^ at a current density of 100, 300, 500, 1000, 2000, and 5000 mA g^−1^ (each for 10 cycles), respectively. For comparison, the CoO-FLs are tested under the same conditions. Their average discharge capacities are 1521, 1361, 1187, 383, and 179 mA h g^−1^, respectively. After increasing the current density from 100 to 5000 mA g^−1^ (60 cycles), the average discharge capacities of CoO-NFs and CoO-FLs stay at 1556 mA h g^−1^ and 1276 mA h g^−1^ when the current density comes back to 100 mA g^−1^. Obviously, both CoO-NFs and CoO-FLs manifest a good rate capability. This may be attributed to the following aspect: the copper foam, acting as an elastic support, provides porous features for the growth of the nanosized CoO, which reduces the exchange resistance of Li^+^ between active materials and electrolytes and shortens the charge-transfer pathway [49].

**Figure 6:**
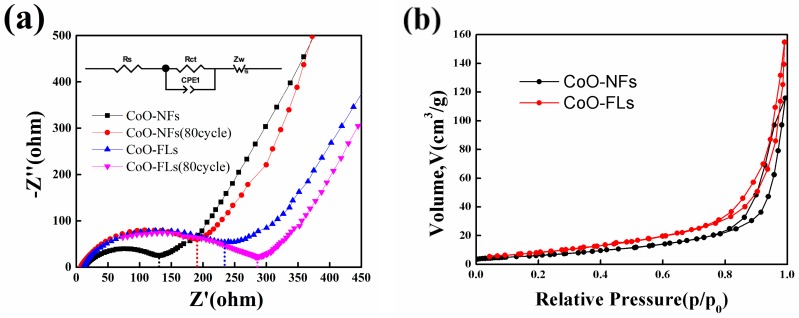
Electrochemical impedance spectra (**a**) and the N2 adsorption/desorption isotherms (**b**) of CoO-NFs and CoO-FLs.

**Figure 7:**
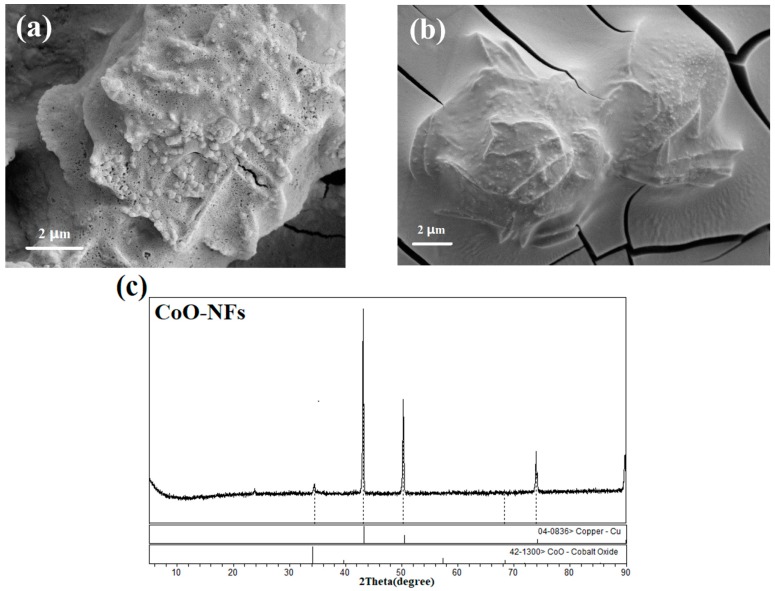

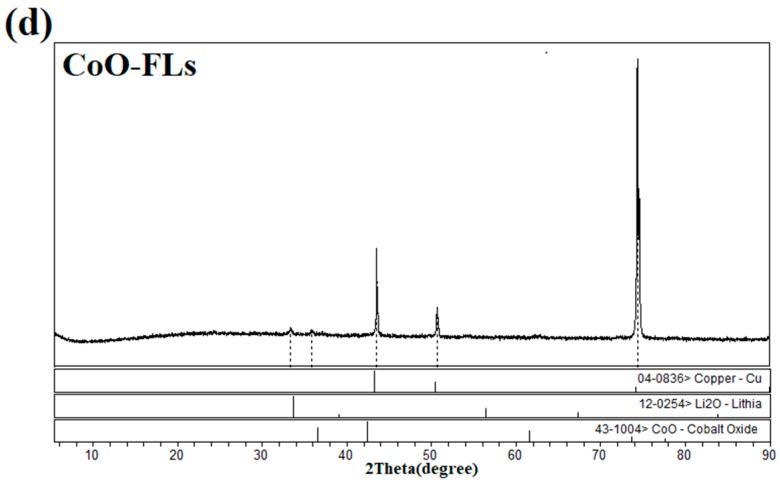
The morphologies and XRD pattern of CoO-NFs electrode (**a**,**c**) and CoO-FLs (**b**,**d**) after 80 cycles.

The comparison of impedance plots of CoO-NFs and CoO-FLs is shown in Figure 6a at a frequency range from 0.01 Hz to 10 kHz. The high-frequency area reflects the bulk cell resistance (Rs), which relates to the electrode and the electrolyte. The existence of the semicircle is usually ascribed to the charge transfer process, while Rct is the charge transfer resistance results from the semicircle diameter. Evidently, the diameter of the semicircle for CoO-NFs is much smaller than that for CoO-FLs, indicating lower charge-transfer impedance in the electrode/electrolyte. CPE1 (constant phase element) is a capacitance element coupled with Rct. The inclined line in the low-frequency area indicates the Warburg impedance associated with the lithium-diffusion process (Zw) [50]. Evidently, the semicircle diameter of the samples in the 80 cycle is correspondingly larger than the samples in the first cycle. This may be ascribed to the structural integrity of the electrodes (displayed in Figure 7a,b) and the thickening of the SEI films when Li^+^ is continuously deposited on the surface [51]. Furthermore, the three-dimensional porous structure of copper foam contributes a large specific surface area of 24.8 m^2^/g for CoO-NFs and 32.8 m^2^/g for CoO-FLs, measured by BET, as shown in Figure 6b. The larger specific surface of CoO-FLs causes the formation of more SEI films and irreversible capacity loss, which may be an explanation for the difference in electrochemical performance between CoO-NFs and CoO-FLs.

Figure 7a,b shows the morphologies of CoO-NFs and CoO-FLs after 80 cycles. It can be seen from Figure 7a that the nanoflakes are staggered, so the specific structure can be retained. It can be seen in Figure 7b that there are many large cracks over the surface, which indicates the destruction of the CoO-FLs’ anode. Furthermore, it can be seen from the XRD patterns (Figure 7c,d) that CoO-NFs shows better crystal perfection than CoO-FLs. The structural damage during the lithium intercalation and deintercalation process is more serious in CoO-FLs, which is consistent with the results of SEM in Figure 7a,b. Compared with CoO-FLs, CoO-NFs exhibit higher reversible capacity and better cycle stability after 80 cycles at the same current densities. This may be attributed to the maintenance of a compact and staggered structure after long-term cycling.

## 4. Conclusions

In summary, two different morphologies of nanosized CoO electrodes are successfully fabricated on a copper foam substrate through the hydrothermal method by adjusting the pH values of solutions. The as-prepared CoO-NFs and CoO-FLs, as anode materials for LIBs, exhibit excellent electrochemical performance. CoO-NFs, in particular, achieved a reversible capacity of 1776 mA h g^−1^ after 80 cycles at 100 mA g^−1^, proving that they are a promising anode material for high-capacity LIBs. This work also provides new insight into the choosing of different current collectors to fabricate high-quality, binder-free electrodes, which can greatly enhance the performance of LIBs.

## Figures and Tables

**Table 1:** Results reported in the literature on the electrochemical performance of CoO anodes.

Materials		Performance		Reference
CoO	nanoflakes	1776 mA h g^−1^ after 80 cycles	At 100 mA g^−1^	This work
CoO	nanowire clusters	1249 mA h g^−1^ after 50 cycles	At 200 mA g^−1^	[33]
CoO	nanowire arrays	1300 mA h g^−1^ after 90 cycles	At 100 mA g^−1^	[31]
CoO	nanosheet arrays	1000 mA h g^−1^ after 100 cycles	At 1000 mA g^−1^	[34]
CoO	semisphere arrays	695 mA h g^−1^ after 150 cycles	At 500 mA g^−1^	[35]
CoO	Cu-doped	800 mA h g^−1^ after 80 cycles	At 500 mA g^−1^	[17]
CoO	nanosheets	637 mA h g^−1^ after 200 cycles	At 100 mA g^−1^	[28]

**Table 2:** The element mass fraction of CoO-NFs and CoO-FLs.

Samples	Co (mg)	Average (mg)
NF-1	2.256	2.021
NF-2	1.785	
FL-1	1.246	1.161
FL-2	1.076	
